# Novel TLR7/8 agonists promote activation of HIV-1 latent reservoirs and human T and NK cells

**DOI:** 10.3389/fmicb.2023.1033448

**Published:** 2023-01-27

**Authors:** Yangyang Li, Zhisong Wang, Ying Hou, Xiaoyu Liu, Junxian Hong, Xuanling Shi, Xiaojie Huang, Tong Zhang, Xuebin Liao, Linqi Zhang

**Affiliations:** ^1^Department of Basic Medical Sciences, School of Medicine, NexVac Research Center, Comprehensive AIDS Research Center, Tsinghua University, Beijing, China; ^2^Key Laboratory of Bioorganic Phosphorus Chemistry and Chemical Biology of Ministry of Education, School of Pharmaceutical Sciences, Tsinghua University, Beijing, China; ^3^Center for Infectious Diseases, Beijing Youan Hospital, Capital Medical University, Beijing, China

**Keywords:** HIV-1, latent reservoir, TLR7 and TLR8 agonists, TNF-α, T cells, NK cells

## Abstract

Antiretroviral therapy can successfully suppress HIV-1 replication to undetectable levels but fails to eliminate latent and persistent HIV-1 reservoirs. Recent studies have focused on the immunomodulatory agents such as Toll-like receptor 7 and 8 (TLR7 and TLR8) capable of activating, thereby rendering the reservoir susceptible to antiretroviral inhibition and immune recognition and elimination. In this context, this study focused on generating a diverse repertoire of TLR7/8 agonists to identify more potent candidates for activating latent HIV-1 and immune cells’ response. Through combinational strategies of computer-aided design and biological characterization, 159 pyrido [3,2-d] pyrimidine and pyridine-2-amine-based derivatives were synthesized. Of which, two TLR7/8 dual and one TLR8-specific agonists with exceptionally high potency in activating HIV-1 latent reservoirs in cell lines and PBMCs of patients with persistent and durable virologic controls were identified. Particularly, these agonists appeared to enhance NK and T cells activity, which were correlated with the degree of surface activation markers. The outcome of this study highlights the remarkable potential of TLR7/8 agonists in simultaneously activating HIV-1 from the latently infected cells and augmenting immune effector cells.

## Introduction

In the past 40 years, the acquired immunodeficiency syndrome (AIDS) caused by human immunodeficiency virus type 1 (HIV-1) has become one of the most deadly infectious diseases in the world, posing a huge threat to global health (Ghosn et al., 2018). Although highly active antiretroviral therapy (HAART) can achieve sustained suppression and reduce transmission of HIV-1, it requires lifelong treatment and fails to cure the patients completely (Finzi et al., 1999; Zhang et al., 1999). The major barrier lies in the existence of a latent virus reservoir that contains replication-competent but transcriptionally dormant HIV DNA proviruses (Finzi et al., 1997; Wong et al., 1997; Bachmann et al., 2019). However, once the HAART is interrupted, the latent proviruses become activated and viral RNA is produced in the plasma up to the pre-treatment levels. Long-lived resting memory CD4+ T cells are the best-characterized cell type harboring the latent HIV-1, although monocytes and macrophages also contribute to the latent pool (Delobel et al., 2005; Gibellini et al., 2008; Clayton et al., 2017; Kruize and Kootstra, 2019). So far, cellular markers specific for the latently infected cells have not been identified, rendering the cure strategy extremely challenging.

One of the most well-explored strategies to eliminate the latent reservoir is to employ latency-reversing agents (LRAs) to activate, thereby rendering them susceptible to recognition and clearance by immune cells such as NK cells and cytotoxic CD8+ T cells. The LRAs under active investigation include histone deacetylase inhibitors (HDACi), histone methyltransferase inhibitors, bromodomain extra-terminal inhibitors (BETi), protein kinase C (PKC) agonists, as well as cytokines and TLR agonists (Kim et al., 2018). Multiple HDACi, such as vorinostat, romidepsin, and panobinostat, demonstrate capability in activating latency through epigenetic modification of HIV-1 LTR. Their impact in the clinical setting, however, was rather disappointing. While a significant increase in a cell-associated unsliced HIV RNA was noticed, no statistical increase of HIV-1 RNA in plasma and reduction of total HIV DNA was detected in any of the reported studies (Elliott et al., 2014; Rasmussen et al., 2014; McMahon et al., 2020).

Recent studies have focused intensively on the immunomodulatory LRAs, including Toll-like receptor (TLR) agonists, immune-checkpoint inhibitors, and cytokines. These novel LRAs possess the advantage of activating latent reservoirs and enhancing immune cell activity (Del Prete et al., 2019; Harper et al., 2020; McBrien et al., 2020). TLRs are a family of pattern recognition receptors (PRRs) involved in sensing the pathogen-associated molecular patterns by the innate immune system during early infection (Aderem and Ulevitch, 2000; Takeuchi and Akira, 2010). A total of 10 functional TLRs have so far been identified in humans, in which TLR3, TLR7, TLR8, and TLR9 are expressed on the intracellular endosomal membrane and are commonly involved in recognizing nucleic acids (Kanzler et al., 2007; Kawai and Akira, 2010; Fitzgerald and Kagan, 2020). TLR7 and TLR8 have a similar dimer structure but distribute differently on diverse cells. While TLR7 is largely distributed in the plasmacytoid dendritic cells (pDCs) and B cells, TLR8 exists in the monocytes, macrophages, and myeloid dendritic cells (mDCs) (Ito et al., 2002). Activation of TLR7 and TLR8 recruit MyD88, but TLR7 predominantly activates downstream signaling through IRF7 to induce anti-viral type 1 interferon, whereas TLR8, through transcription factor NF-κB, induces the proinflammatory cytokines (Beignon et al., 2005; Kawai and Akira, 2010). Several TLR7/8 agonists have been characterized to reverse HIV-1 latency *in vitro* and *in vivo*. For instance, GS9620 is a potent TLR7-specific agonist synthesized by Gilead Sciences and is in progress in phase 2 clinical trials. GS9620 induced transient viremia and reduced the latent reservoirs in SIV-infected rhesus macaques on ART (Lim et al., 2018). GS9620 combined with HIV-1 broadly-neutralizing antibody (bNAb) PGT121 delayed viral rebound in SHIV-infected monkeys when ART was interrupted (Borducchi et al., 2018). The results of phase 1b illustrate some decrease of intact proviral DNA in patients treated with oral GS9620, and the viral rebound time increased from 4.1 to 5.1 weeks compared with the placebo group (SenGupta et al., 2021). Similarly, a few TLR8 agonists have also been found to activate HIV-1 latency in CD4+ T cell line J-Lat and human peripheral mononuclear cells (Schlaepfer and Speck, 2011; Rochat et al., 2017). More importantly, TLR7/8 agonists enhanced NK and T cells mediated killing, making this class of LRAs promising candidates for achieving a functional cure for HIV-1 infection (Hart et al., 2005).

Capitalizing on the progress made in TLR7/8 as LRAs, this study aims to expand and identify more potent TLR7/8 agonists in activating the HIV-1 reservoir and enhancing cellular immunity against HIV-1 latency. Using combination approaches in computer-aided design and biological characterization, 159 pyrido [3,2-d] pyrimidine and pyridine-2-amine-based derivatives were synthesized. We identified two TLR7/8 dual (D018 and C142) and one TLR8-specific (B-130a) agonists with exceptionally high potency in activating HIV-1 latent reservoirs in cell lines and PBMCs of patients with persistent and durable virologic controls. Moreover, these agonists appear to induce immune effector cell activation, such as NK cells and T cells. Collectively, the results of this study illustrate the promising role of TLR7/8 agonists as potential LRAs in simultaneously increasing HIV-1 expression from the latently infected cells as well as augmenting immune cells’ response.

## Results

### Design and characterization of novel TLR7/8 dual and TLR8-specific agonists

**Figure 1:**
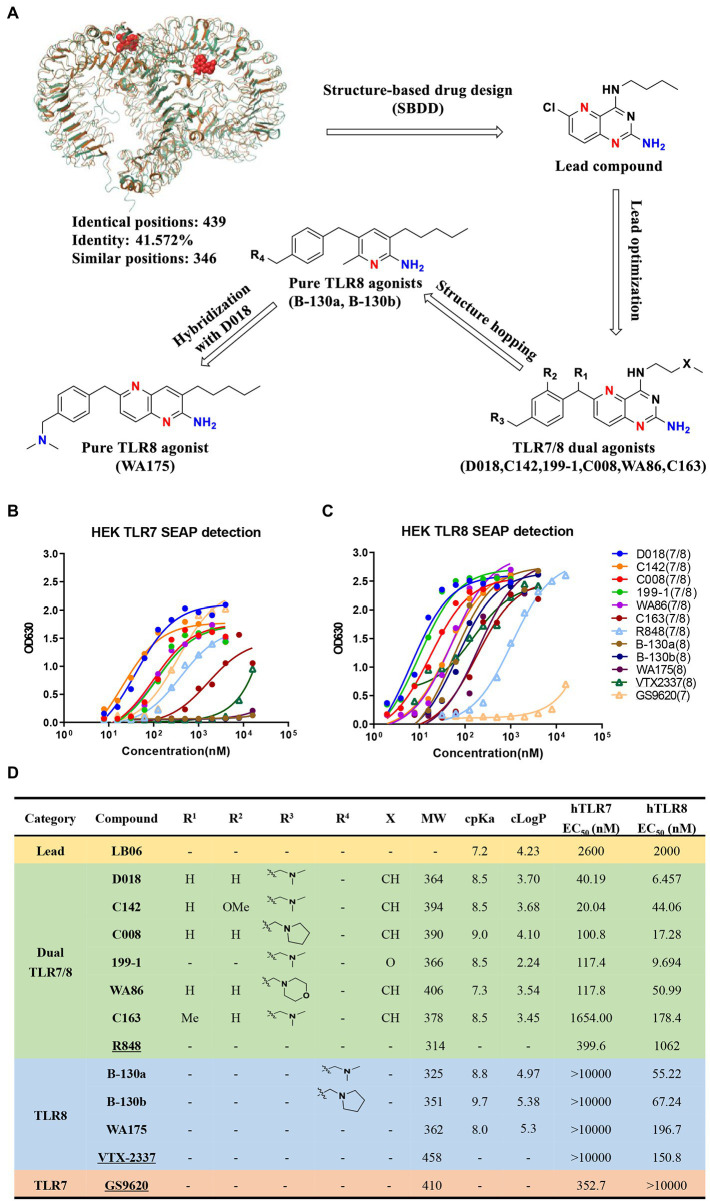
Design, synthesis, and evaluation of novel TLR7/8 agonists through HEK-Blue cell lines expressing either hTLR7 or hTLR8 receptor. **(A)** The design flowchart and chemical structures of novel TLR7/8 agonists with single TLR8 or dual TLR7/8 agonistic activity. Superimposition of MmTLR7-R848 cocrystal (5GMH, brown) and hTLR8-R848 cocrystal (3W3N, bottle green) was performed by Maestro software (v9.1). **(B,C)** Activity evaluation of TLR7/8 agonists through HEK-Blue cell lines expressing either hTLR7 **(B)** or hTLR8 **(C)** receptor, measured by the secreted embryonic alkaline phosphatase (SEAP) in the culture supernatant. Commercially available GS9620 (TLR7 agonist), VTX-2337 (TLR8 agonist), and R848 (TLR7/8 dual agonist) labeled unfilled triangle are reference compounds. **(D)** TLR7/8 agonists were divided into 4 categories, exhibiting the molecular structure (R1,R2,R3,R4,X refer to the functional group in **A**), molecular weight (MW), cpKa, cLogP, as well as EC50 values for hTLR7 and hTLR8. Commercially available GS9620 (TLR7 agonist), VTX-2337 (TLR8 agonist), and R848 (TLR7/8 dual agonist) labeled as underlined are reference compounds.

Based on the extensive analysis of the reported crystal structure of TLR7 and TLR8 and the structure–activity relationship (SAR) of the associated small molecule agonists (Tanji et al., 2013; Zhang et al., 2016), a total of 160 compounds were designed, synthesized and characterized to obtain novel and more potent TLR7/8 dual and TLR8-specific agonists, as shown in the flow chart (Figure 1A). Starting with the lead compound, 76 pyrido [3,2-d] pyrimidine derivatives were synthesized and optimized through structural modification from the deep hydrophobic region to the solvent-exposed region. Seventy-six compounds were evaluated for their *in vitro* activity using HEK-Blue hTLR7 or hTLR8 reporter cells, stably expressing the human TLR7 or TLR8 and an NF-κB/AP1-inducible SEAP (secreted embryonic alkaline phosphatase reporter gene). We identified six compounds, D018, C008, 199–1, WA86, C142, and C163, that could induce substantial levels of SEAP in the culture supernatant of both HEK-Blue hTLR7 and hTLR8 cells and therefore classified as TLR7/8 dual agonists. D018 and C142 exhibited the most potent agonistic activity, while the remaining four were rather varied but invariably stronger than the relevant control R848 except C163 (Figures 1B–D). To our knowledge, these molecules are the most potent TLR7/8 dual agonists reported so far.

Following this, using D018, 83 additional pyridine-2-amine-based derivatives were designed and synthesized to achieve high selectivity for TLR8 using the scaffold hopping strategy. By evaluating these compounds using the same *in vitro* activity, two candidate compounds, B-130a and B130b were identified, demonstrating high selectivity for TLR8 over TLR7. The agonistic activity toward TLR8 was 55.22 nM and 67.24 nM, respectively, for B-130a and B130b, while that toward TLR7 was all above 10,000 nM (Figures 1B–D). An attempt was made further to improve TLR8 activity and specificity by generating a series of D018-B-130a hybrids. However, only one compound, WA175, showed moderate activity with an EC50 of 196.7 nM. Nevertheless, two TLR-8-specific compounds, B-130a and B130b, were superior to the control VTX-2337 (Figures 1B–D).

The physicochemical properties of these novel TLR7/8 dual and TLR8-specific agonists were also evaluated through ACD/Percepta platform and Molinspiration software. As shown in Figure 1D, all these compounds demonstrate moderate basicity (cpKa >7.0), consistent with our intended design and SAR analysis, where introducing a basic group was associated with enhancing agonistic activity. Moreover, all these compounds except B-130b and WA175 had acceptable lipophilicity (cLogP) less than 5, obeying Lipinski’s rule of five (Figure 1D). Then, the three most potent agonists, two TLR7/8 dual compounds, D018(TLR7/8) and C142(TLR7/8) and one TLR8-specific compound, B-130a (TLR8), were selected to analyze their potential in activating HIV-1 latent reservoirs and compared with the commercially available agonists R848 (TLR7/8), VTX-2337 (TLR8), and GS9620 (TLR7) (Supplementary Figure S1).

### TLR7/8 agonists activated latent HIV-1 and induced inflammatory cytokines from PBMCs of well-suppressed patients

**Figure 2:**
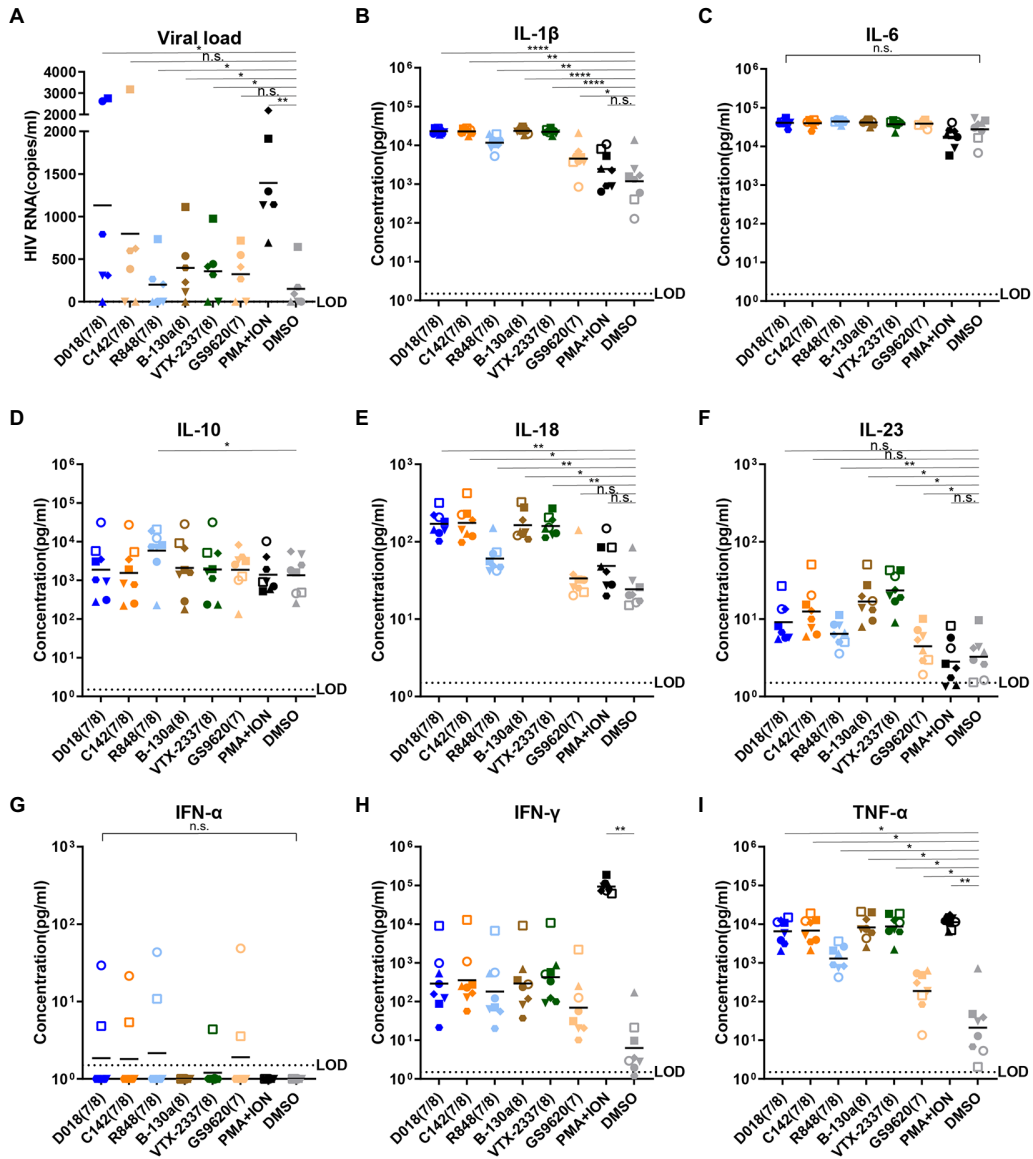
TLR7/8 agonists activate latent HIV-1 and inflammatory cytokines from PBMCs of infected individuals. **(A)** HIV-1 RNA copies in the supernatant were measured by RT-qPCR 4 days after stimulation (*n* = 6). **(B–I)** Concentrations of inflammatory cytokines in the culture supernatant 2 days after stimulation by TLR7/8 agonists, including IL-1β **(B)**, IL-6 **(C)**, IL-10 **(D)**, IL-18 **(E)**, IL-23 **(F)**, IFN-α **(G)**, IFN-γ **(H)**, and TNF-α **(I)**. DMSO was negative control whereas PMA plus Ionomycin were positive control. PBMC from each HIV-1 infected patient (*n* = 6) with different shape was filled and PBMC from each normal control (*n* = 2) with different shape was an open symbol. Data are shown as the mean of two replicates for each donor. Statistical difference between various TLR7/8 agonists and DMSO group analyzed by paired-*t*-test. ****p < 0.0001, ***p < 0.001, **p < 0.01, *p < 0.05, n.s. not significant.

To study the potential of novel TLR7/8 in activating latent HIV-1 reservoir, TLR7/8 dual agonists D018 and C142 and TLR8-specific agonist B-130a were mixed with PBMCs from six well-suppressed patients whose viral load in the plasma was less than 50 copies/ml, and CD4 counts were more than 350 cells/ml for more than 6 years. After incubation at 37°C for 96 h, the supernatant was collected and analyzed for HIV-1 RNA copies and compared with those stimulated by commercially available agonists, TLR7/8 dual agonist R848, TLR8 specific agonist VTX-2337, and TLR7 specific agonist GS9620, as well as the positive control phorbol myristate acetate (PMA) plus ionomycin and the negative control DMSO was used as a solvent for these agents. As shown in Figure 2A, dual TLR7/8 agonists D018 and C142 and TLR8 specific agonist B-130a activated detectable levels of HIV-1 RNA copies in the supernatant. D018 was the most potent, followed by C142 and then B-130a. The commercially available R848, VTX-2337, and GS9620 showed similar potency as B-130a but substantially lower than D018 and C142. The positive control PMA combined with ionomycin stimulated the highest levels of HIV-1 RNA copies in the supernatant, while the solvent DMSO was the lowest (Figure 2A).

Then, a total of 13 human inflammatory cytokines and chemokines (IL-1β, IFN-α2, IFN-γ, TNF-α, CCL2, IL-6, CXCL8, IL-10, IL-12p70, IL-17A, IL-18, IL-23, and IL-33) were measured in the culture supernatant 48 h after stimulation and compared with two healthy donors. Only 8 out of 13 became detectable and largely fell into the proinflammatory cytokines (IL-1β, IL-6, IL-18, IL-23, and TNF-α), Th1-and Th2-type cytokines (IL-10 and IFN-γ), and type I (IFN-α2) and type II (IFN-γ) interferons (Figures 2B–I). TLR7/8 dual agonists D018 and C142 demonstrated similar potency in triggering the release of these cytokines and were either equivalent (IL-6, IL-10, IL-23, and IFN-γ) or higher (IL-1β, IL-18, and TNF-α) than the commercially available R848. TLR8-specific B-130a and commercially available VTX-2337 exhibited comparable potency but were invariably higher than TLR7-specific GS9620 and negative control DSMO. Compared to DSMO, positive control PMA/ionomycin showed a substantially stronger impact on IFN-γ and TNF-α. At the same time, the differences for the remaining cytokines were minimal, suggesting that DMSO solvent alone could also trigger some release of these cytokines (Figures 2B–I). Interestingly, while no substantial differences were found for most of the cytokines tested, a clear trend of higher levels of IFN-α2 and IFN-γ in healthy individuals than in the HIV-1 infected indicates distinct differences in host innate immune responses between the two groups of individuals toward the tested agonists. These results imply that novel TLR7/8 dual agonists D018 and C142 and TLR8-specific agonist B-130a could activate HIV-1 latent reservoir and cytokine release in a higher capacity than the commercially available R848 and VTX-2337 counterparts.

### TLR7/8 agonists activate latent HIV-1 in the U1 monocytic cell lines through the TLR8 pathway

**Figure 3:**
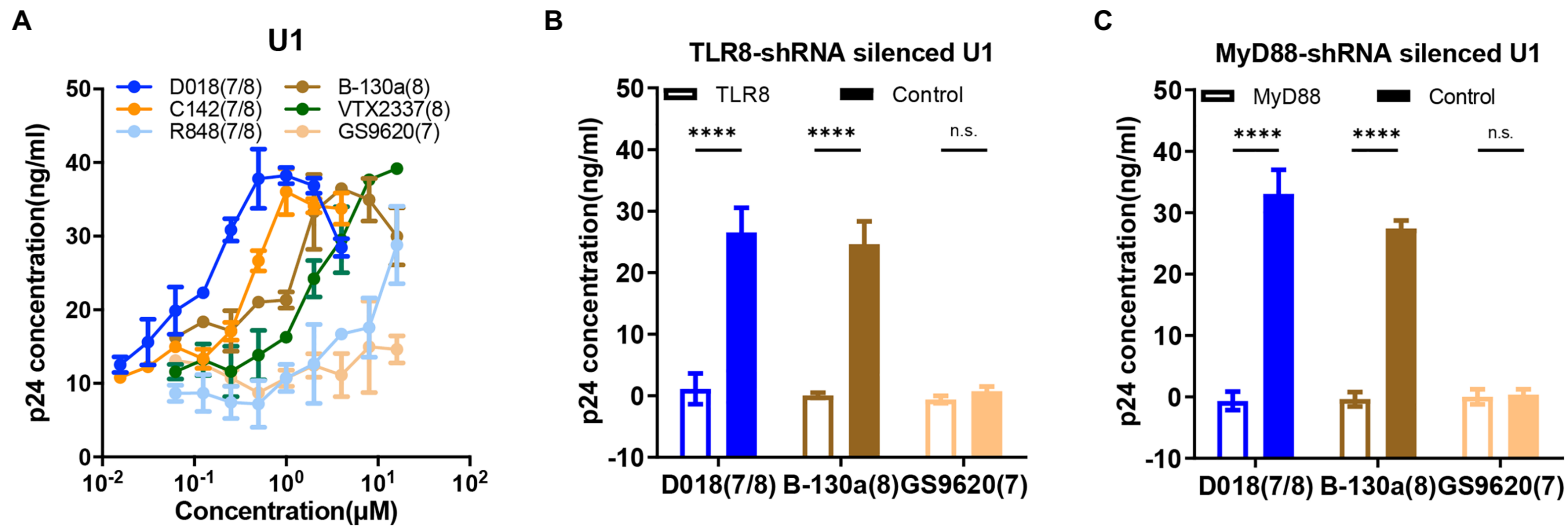
TLR7/8 agonists directly activate latent HIV-1 from monocytic U1 cell line. **(A)** Dose–response curves for HIV-1 activation in U1 cell line by the selected novel and control TLR7/8 agonists. Data are shown as mean ± SD (*n* = 4). **(B,C)** The p24 concentration in the supernatant of U1 cell line stimulated by D018 (TLR7/8), B-130a (TLR8), or GS9620 (TLR8) before and after **(B)** TLR8-shRNA silencing or **(C)** MyD88-shRNA silencing compared with control shRNA silencing. Data are shown as mean ± SD (*n* = 4). Statistical difference between various TLR7/8 agonists was analyzed by two-way ANOVA. ****p < 0.0001, ***p < 0.001, **p < 0.01, *p < 0.05, n.s. not significant.

HIV-1 latently infected cell line U1 was used further to study the potential activation mechanism by TLR7/8 agonists. U1 was a promonocyte cell line generated by chronic infection with HIV-1 into U937. A serial dilution of D018, C142, and B-130a agonists and commercially available R848, VTX-2337, and GS9620 were mixed with U1 and monitored for production of HIV-1 antigen p24 in the supernatant by ELISA. As shown in Figure 3A, D018 was the most potent in stimulating p24 production with an EC50 of 70 nM, followed by C142 and then B-130a. Commercially available VTX-2337 and R848 also induced p24 production, albeit at lower efficiency. GS9620, however, failed to trigger detectable levels of p24 even at the highest concentration of 16 μM (Figure 3A).

To gain deeper insight into the mechanisms of activating HIV-1 latency by TLR7/8 agonists, a series of knockdown experiments were conducted in the U1 cell line using a short hairpin RNA (shRNA) lentivirus technology. As GS9620 failed to activate p24 production (Figure 3A), the TLR8 pathway is largely believed to mediate the agonistic effect of TLR7/8. Hence, TLR8-knockdown, MyD88-knockdown, and sham-knockdown U1 cell lines were generated using shRNA lentiviruses, and the silencing effect was verified by qPCR (Supplementary Figures S2A,B) and western-blot analysis (Supplementary Figure S2C). When exposed to D018, B-130a, and GS9620 at a final concentration of 1 μM, knockdown of TLR8 or MyD88 expression essentially abolished the p24 production compared to the sham-knockdown U1 cell line (Figures 3B,C). As shown above, GS9620 could not induce detectable levels of p24 in the supernatant of sham-knockdown or original U1 cell lines. The expression levels of downstream molecules nuclear p65 and phosphorylated p65-s536 in the original U1 cells were further measured through western-blot, as earlier studies indicated that the activation of TLR8 ultimately led to the NF-κB signaling to regulate the expression of a broad range of genes including cytokines (Gringhuis et al., 2010). After incubation at 37°C for 30 min, the U1 cells stimulated by D018 and B-130a exhibited a significant increase in nuclear phos-p65-S536, while that stimulated by GS9620 was similar to the negative control DMSO (Supplementary Figure S2D). These results confirmed the hypothesis that activating latent HIV-1 in the U1 cell line by the novel TLR7/8 agonists were mediated through TLR8 and downstream MyD88 and ultimately regulated through NF-κB signaling.

### TLR7/8 agonist effect is mediated through soluble TNF-α in the J-LAT T cell lines

**Figure 4:**
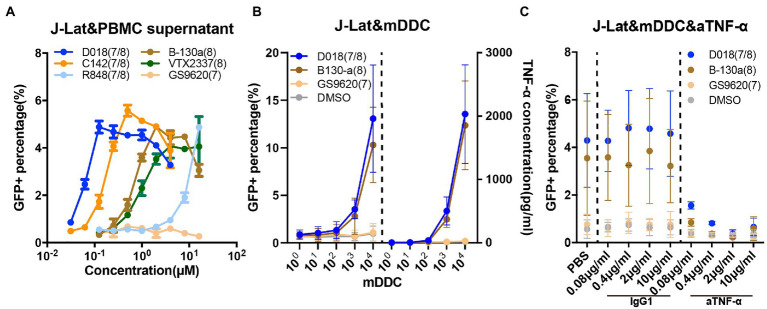
TLR7/8 agonists indirectly activate latent HIV-1 from the J-Lat CD4 T cell line through TNF-α. **(A)** Dose–response curves for HIV-1 activation in J-Lat CD4 T cell line by the selected novel and control TLR7/8 agonists. The GFP+ percentage indicated the proportion of J-Lat activated. Data are shown as mean ± SD of 2 independent experiments). **(B)** Dose–response curves for HIV-1 activation and TNF-α release in the J-Lat and mDDC co-culture system 48 h after stimulation by D018, B130-a, GS9620, and DMSO. Data are shown as mean ± SD (*n* = 4). **(C)** Dose–response curves for HIV-1 activation of J-Lat CD4 T cells with varying concentrations of anti-TNF-α monoclonal antibody compared with that of irrelevant IgG1 antibody control, 48 h after stimulation by D018, B130-a, GS9620, and DMSO. The GFP+ percentage indicated the proportion of J-Lat activated. Data are shown as mean ± SD (*n* = 4).

J-LAT is a Jurkat-based CD4 T cell line with an integrated latent HIV-1 provirus in which GFP replaces the nef coding sequence, and the env is non-functional due to a frameshift (Jordan et al., 2003). All the testing agonists failed to induce detectable GFP-positive J-LAT cells through direct stimulation (Supplementary Figure S3A). In contrast, when J-LAT cells were incubated with the supernatant from healthy donor PBMCs stimulated with the testing agonists for 48 h, a substantial proportion of GFP-positive cells became detectable dose-dependently (Figure 4A). Supernatant from D018-stimulated PBMCs was the most potent in activating GFP-positive cells, followed by C142 and B-130a. VTX-2337 demonstrated similar potency to B-130a but was substantially stronger than R848. GS9620 stimulated supernatant did not show any detectable effect on activating GFP expression in J-LAT cells, even at the highest concentration of 16 μM. These results indicate that our novel agonists can activate latent HIV-1 in J-LAT T cells, but only indirectly through some soluble molecules in the supernatant of stimulated PBMCs.

Previous experiments demonstrated that the activation of HIV-1 from J-LAT cells was mediated through soluble TNF-α released by the monocyte-derived dendritic cells (MDDCs) (Schlaepfer and Speck, 2011). TLR7/8 dual agonists and TLR8 specific agonists could induce PBMC to express high levels of TNF-α, while TLR7 specific agonist only could induce low levels of TNF-α but high IFN-α (Supplementary Figures 3B,C). Then, J-LAT cells were stimulated with different dilutions of soluble TNF-α and IFN-α. Only TNF-α could activate GFP expression in J-Lat cells in a concentration gradient, while IFN-α failed to activate J-LAT cells even at 10 ng/ml (Supplementary Figure S3D). Therefore, we focused on this possibility to explore the mechanism of activation of latent HIV-1 of our novel TLR7/8 agonists. We sorted CD14+ monocytes from the PBMC of a healthy individual and differentiated them into MDDCs through stimulation with GM-CSF and IL-4. The expression of hTLR7 and hTLR8 was more than 10-fold higher in MDDCs than in the J-LAT cells by qPCR (Supplementary Figure S3E). A serial number of differentiated MDDCs were co-cultured with J-Lat cells in the presence of TLR7/8 agonists at 1 μM for 48 h. As shown in Figure 4B, when D018 and B-130a were added into the co-culture, a marked increase in the percentage of GFP+ J-LAT cells and a concomitant increase in the soluble concentration of TNF-α were observed. In co-cultures with GS9620 or the negative control DMSO, no detectable levels of GFP+ J-LAT cells or soluble TNF-α were found. Furthermore, neutralizing TNF-α with antibodies resulted in a dose-dependent decrease in GFP+ J-LAT cells, while an irrelevant IgG1 antibody exhibited a minimal effect (Figure 4C). This is consistent with previous reports and confirmed our hypothesis that soluble TNF-α released by the MDDCs largely mediated the activation of latent HIV-1 by TLR7/8 agonists in J-LAT cells. In other words, the activation of TLR7/8 agonists on latent HIV-1 was indirect in the J-LAT cell line, while U1 was direct through the TLR8 pathway.

### TLR7/8 agonists activate latently infected PBMCs indirectly through soluble TNF-α

The mechanism of TLR7/8 agonist activation of the latent reservoir in PBMCs derived from HIV-1 chronically infected patients was further investigated. It has been reported that most of the latently infected cell types are resting memory CD4 T cells, with a very small proportion of monocytes and dendritic cells (Finzi et al., 1999; Gibellini et al., 2008). Previous results in HIV-1 latently infected cell lines showed that TLR7/8 agonists alone could not directly stimulate latently infected CD4 T cell lines (Supplementary Figure S3A).

CD4 T cells derived from HIV-1 well-suppressed patients were then stimulated with D018, B-130a, GS9620, positive control PMA + ION, and negative control DMSO. Supplementary Figures S4A,B show that the positive control significantly stimulated the transcriptional expression of HIV-1 RNA copies in the supernatant and p24 in CD4 T compared to the negative control DMSO. However, none of the three TLR7/8 agonists detected the ability to activate HIV-1 latently infected CD4 T cells.

**Figure 5:**
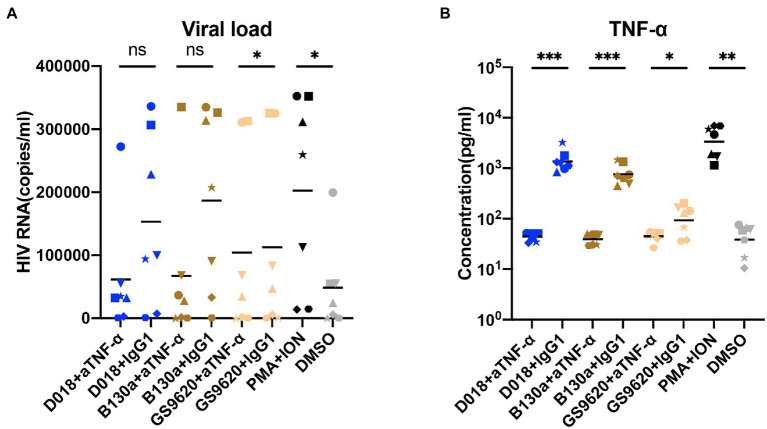
TLR7/8 agonists indirectly activate latent-infected cells derived from HIV-1 positive patients through TNF-α. **(A,B)** The PBMCs derived from HIV-1 viremic patients were stimulated by D018 (TLR7/8), B-130a (TLR8), GS9620 (TLR8) with anti-TNF-α monoclonal antibody compared with that of irrelevant IgG1 antibody control. **(A)** The HIV-1 RNA copies and **(B)** TNF-α concentrations in the supernatant were measured after stimulation. Data are shown as mean ± SD (*n* = 7). Statistical difference was analyzed by paired *t*-test. ****p < 0.0001, ***p < 0.001, **p < 0.01, *p < 0.05, n.s. not significant.

TLR7/8 agonists activated PBMCs from seven well-suppressed patients with the addition of anti-TNF-α monoclonal antibody or IgG1-control monoclonal antibody, respectively. D018, B-130a, and the positive control PMA + ION increased the levels of HIV-1 RNA copies in the supernatant and the concentration of TNF-α concomitantly. In co-cultures with GS9620 or the negative control DMSO, low detectable levels of HIV-1 RNA copies or soluble TNF-α were found. Furthermore, neutralizing TNF-α with antibodies significantly blocked the activity of latent HIV-1 by TLR7/8 agonists, which is consistent with the results of TLR7/8 agonists activating latently infected cell lines. These results confirmed that TNF-α plays a critical role in the indirect stimulation of HIV-1 latently infected PBMC by TLR7/8 agonists (Figures 5A,B).

### TLR7/8 agonists preferentially activate NK and T cells

**Figure 6:**
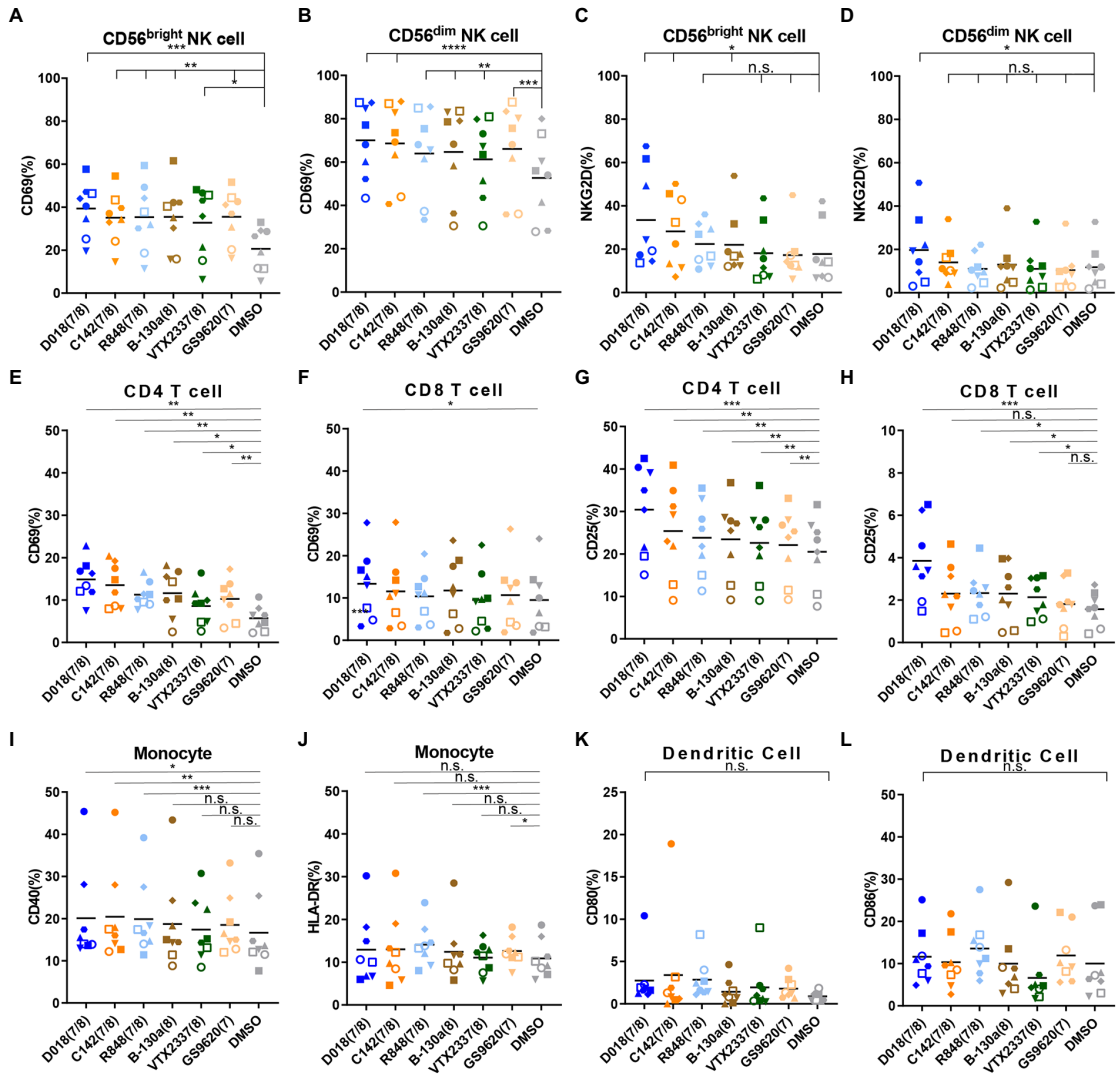
TLR7/8 agonists prefer to induce the NK cells and T cells activation and IFN-γ expression. **(A–D)** PBMCs from healthy donors were stimulated by different TLR7/8 agonists or DMSO for 24 h, and different activated markers in several immune cells were analyzed by flow cytometry. The percentage of **(A)** CD69 positive CD56^bright^ NK cells and **(B)** CD56^dim^ NK cells, **(C)** NKG2D positive CD56^bright^ NK cells, and **(D)** CD56^dim^ NK cells. **(E–H)** The percentage of **(E)** CD69 positive CD4 T cells and **(F)** CD8 T cells, **(G)** CD25 positive CD4 T cells, and **(H)** CD8 T cells. **(I,J)** The percentage of **(I)** CD40 positive and **(J)** HLA-DR positive monocytes. **(K,L)** The percentage of **(K)** CD80 positive and **(L)** CD86 positive dendritic cells. Each different shaped point represents one donor and data is shown as the mean of 8 donors. Statistical difference between various TLR7/8 agonists and DMSO group was analyzed by paired *t*-test. ****p < 0.0001, ***p < 0.001, **p < 0.01, *p < 0.05, n.s. not significant.

TLR family is essential in connecting innate immunity with acquired immunity in fighting against infection. It was then studied whether the identified TLR7/8 agonists could activate cell populations within PBMC, thereby potentiating their effector functions. PBMCs from healthy donors were stimulated by TLR7/8 agonists for 24 h and analyzed for several activation markers by flow cytometry. The representative diagram of the gating strategy for each immune cell is shown in Supplementary Figure S5. All tested TLR7/8 agonists activated CD69 and NKG2D expression on CD56^bright^ and CD56^dim^ NK cells (Figures 6A–D), as well as CD69 and CD25 expression on CD4+ and CD8+ T cells (Figures 6E–H). However, they failed to have the detectable effect of CD40 and HLA-DR expression on monocytes and CD80 and CD86 expression dendritic cells (Figures 6I–L). In almost all instances, the dual TLR7/8 agonist D018 was the most potent, followed by C142, while the B-130a and other commercially available agonist controls were relatively weaker. These results signify that the dual TLR7/8 agonists D018 and C142 can induce the expression of various activation markers for NK and T cells but not for monocytes and dendritic cells.

## Discussion

TLR7 and TLR8 agonists are among the promising agents capable of activating and reversing HIV-1 latent reservoirs in patients treated with antiretroviral therapy and achieving sustained viral suppression. Using combination approaches of computer-aided design and biological characterization, two TLR7/8 dual (D018 and C142) and one TLR8-specific (B-130a) agonists were identified with exceptionally high potency in activating HIV-1 latent reservoirs from U1 and J-LAT6.3 cell lines as well as from PBMCs of patients with persistent and durable virologic controls. The activation mechanism appears to be direct through the TLR8-signaling pathway in the U1 cell line while indirect in the J-LAT6.3 cell line through soluble TNF-α released by the monocyte-derived dendritic cells (MDDCs). The indirect mechanism of soluble TNF-α contributes to the activation in PBMCs of patients, but the exact activation mechanism remains uncertain (Schlaepfer and Speck, 2008; Tsai et al., 2017). This hypothesis is supported by the capacity of these agonists to trigger the expression of various activation markers on NK and T cells and release inflammatory cytokines and chemokines in the supernatant of activated PBMCs. Taken together, these results highlight the remarkable potential of the TLR7/8 agonists in simultaneously increasing the expression of HIV-1 from the latently infected cells and activating the immune cells.

**Figure 7:**
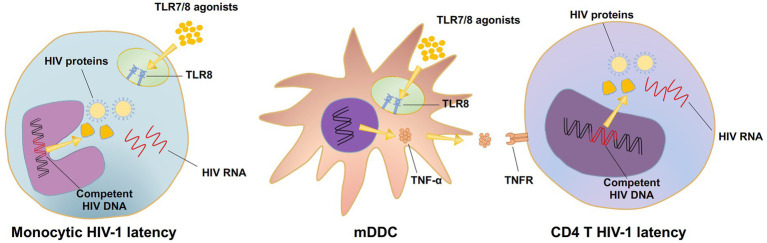
The mechanism diagram of TLR7/8 agonists activating HIV latent reservoirs.

A couple of points need to be highlighted here. The TLR7/8 dual agonists D018 and C142 appeared to be more potent than TLR8-specific B-130a in activating the HIV-1 latent reservoir in PBMC as well as in U1 and J-LAT 6.3 cell lines. The underlying mechanisms accounting for such differences, however, are currently unknown. It is possible that the dual agonists could synergistically act on both TLR7 and TLR8 signal pathways either on the target cells harboring the HIV-1 latent reservoir or on cell types that release more cytokines to promote HIV-1 expression (Schlaepfer et al., 2006). TLR8-specific agonists, on the other hand, could only trigger HIV-1 expression through the TLR8 signal pathway (Rochat et al., 2017). However, this hypothesis does not explain why the control TLR7-specific GS9620 failed to activate HIV-1 expression in U1 and J-LAT6.3 cell lines (Figures 3A, 4A) while exerting some activation levels in PBMCs (Figure 2A). This could be due to the lack of TLR7 expression on U1 cells and relatively lower levels of soluble TNF-α released by the monocyte-derived dendritic cells (MDDCs) after GS9620 stimulation (Figure 2I; Schlaepfer and Speck, 2011; Macedo et al., 2018). In the context of PBMCs, apart from TNF-α, other cytokines, such as IFN-α, may also contribute to the indirect activation of HIV-1 harboring cells, as demonstrated by Tsai and colleagues (Tsai et al., 2017). These results show that TLR7/8 dual and TLR8 specific agonists can induce HIV-1 latency reversal. However, this activity requires sufficient levels of TLR7/8 expression in the targeted cell types, such as HIV-1 latently infected cells or cytokine-releasing cells, to promote HIV-1 activation (Figure 7).

TLR7/8 dual agonist D018 and TLR8-specific agonist B-130a demonstrated higher potency in inducing inflammatory cytokines release than TLR7-specific agonist GS9620. Although the cytokine release may be important in activating latent reservoirs or stimulating immune effector cells, systemic administration may cause side effects such as inflammatory factor storms. Therefore, their safety should be more strictly evaluated. Currently, TLR7/8 agonists are mainly administered topically or orally. Further modification of novel TLR7/8 dual agonist D018 and TLR8-specific agonist B-130a is urged to broaden the therapeutic index as well as conjugation of monoclonal antibodies so that the target delivery of TLR8 agonists could decrease the continual activation to the immune cells and improve the safety *in vivo* (Comeau et al., 2020). Moreover, TLR7/8 dual agonists and TLR8-specific agonists might be sufficient to stimulate NK cell activation. Compared to NK cells, CD4+ and particularly CD8+ T cells appeared less responsive to stimulation by either TLR7/8 dual or TLR8-specific agonists. However, this does not necessarily contradict the findings by Meas et al., where human primary CD4+ T cells express TLR8 and release inflammatory cytokines upon stimulation by TLR8-specific agonist, favoring HIV-1 replication and reversal of latency (Meas et al., 2020).

Overall, this study identified novel TLR7/8 dual and TLR8-specific agonists capable of reversing HIV-1 latency and activating immune cells *ex vivo*. Further studies are required to understand their mechanism of action and potential application for HIV-1 function cure.

## Materials and methods

### Reagents

The TLR7/8 agonists, D018, C008, 199–1, WA86, C142, B-130a, B-130b, WA175, C163 were synthesized by Zhisong Wang in Xuebin Liao’s Lab. R848, GS9620, and VTX2337 were purchased from MedChemExpress (MCE). IL-2, IL-4, GM-CSF, TNF-α and IFN-α were purchased from Sino Biological. BAY 11–7,082 was purchased from Sigma-Aldrich.

### Ethics statement

This study was approved by the Institution Review Board of Tsinghua University. The project number is 20200032. Healthy volunteers over 18 years old who signed the informed consent were recruited to the Tsinghua university hospital for blood donation. Blood sample from HIV-1-infected patients was collected at Beijing YouAn Hospital of Capital Medical University. The project number is EC-B-031-A01-V9.0. All patients were successfully treated by ART (<50 Copies/ml) for more than 6 years, and the CD4 counts were more than 350 cells/ml. Samples from donors were according to protocols approved by the Ethics Committee in Beijing YouAn Hospital of Capital Medical University.

### Cell-lines culture

U1 cells were obtained from the NIH AIDS Reagent Program, and J-LAT 6.3 was kindly provided from The First Hospital of Jilin University, Institute of Virology, and AIDS Research. These cells were cultured in RPMI 1640 medium (Gibco) supplemented with 10% FBS (Gibco) and 1% PS (Macgene). HEK-Blue TLR7 and TLR8 overexpressing cells were purchased from InvivoGen and cultured in DMEM (Gibco) supplemented with 10% FBS, 50 μg/ml Normocin (InvivoGen), 30 μg/ml blasticidin (InvivoGen), and 100 μg/ml Zeocin (InvivoGen). HEK293T cells were purchased from ATCC and cultured in DMEM supplemented with 10%FBS and 1% PS.

### Primary cells isolation, sorting, and culture

PBMCs were isolated from blood samples by density gradient centrifugation using Ficoll-Paque (GE Healthcare). PBMCs were treated with ACK lysis buffer (Gibco) and washed twice in PBS. Then the cells were resuspended in complete RPMI 1640 (Gibco) medium with the addition of 10% Fetal bovine serum (FBS, Gibco), 1% PS (Macgene),1% Glumax (Gibco),1% NEAA (Gibco),1% Sodium pyruvate (Gibco) and incubated at 37°C in 5% CO2.

Monocytes were isolated by CD14 magnetic beads (Miltenyi) according to the manufacturer’s instructions. MDDCs were generated by stimulating monocytes with rhIL-4 and rhGM-CSF (Beyotime) for 5–7 days.

### HEK SEAP detection

Add 20 μl various concentrations of TLR agonists (10 dilutions) and in a 96-well plate. Then HEK-Blue hTLR7 and hTLR8 cells were suspended to 4 × 105 cells/ml in HEK-Blue Detection (InvivoGen) medium and added 180 μl per well. The plates were incubated at 37°C in 5% CO2 for 12 ~ 16 h. Stimulation with a hTLR7or hTLR8 ligand activates NF-κB to induce the production of secreted embryonic alkaline phosphatase (SEAP) in the culture supernatant. SEAP could be measured at OD630nm on an ELISA plate reader.

### Viral production and infection

For generating the TLR8, MyD88 silenced U1 cells, we transfected shRNA lentivirus vector pLKO-TLR8/MyD88/Control shRNA, psPAX2, and pM2D.G into HEK293T cells to produce shRNA lentiviral particle. U1 cells were transduced by shRNA lentivirus overnight with 8 μg/ml polybrene. The cells were replaced with the medium supplemented with 8 μg/ml puromycin (Sigma) to select the positive colonies for 7 days, then were changed with 10% FBS 1640 medium containing 2 μg/ml puromycin to keep. All the shRNA lentivirus vectors were purchased from Sigma.

### RNA extraction and qPCR

To measure the relative expression of TLR7, TLR8, and MyD88, RNA was extracted from 5 × 10^5^ cells using AxyPrep Multisource RNA Miniprep Kit (Axygen) and measured by Nano-drop. An equal amount of RNA was reverse-transcribed into cDNA through the iScript cDNA Synthesis Kit (Bio-red). Samples were quantified by PCR with SYBR Green (Applied Biosystem). All specific primers used for the analysis were designed by qPrimerDB (Lu et al., 2018), and the sequences were shown as follows:

TLR7-F: TCAGCGTCTAATATCACCAGAC,
TLR7-R: CACTGTCTTTTTGCTAAGCTGT,
TLR8-F: TGGCTCACCATTTGTTTTACTG,
TLR8-R: AAAGGAGAACGTTTTTGTCTCG,
MyD88-F: GCGGGCATCACCACACTT,
MyD88-R: TCCGGCGGCACCTCTTTT,
GADPH-F: CCATGTTCGTCATGGGTGTG,
GADPH-R: GGTGCTAAGCAGTTGGTGGTG.

To measure the HIV viral load, viral RNA from supernatants was extracted by MagaBio Plus Viral DNA / RNA Purification Kit (BIOER). The reverse-transcription and analysis of RNA copies were performed using the HIV-1 Real-time PCR detection kit (BIOER).

### HIV p24 antigen ELISA detection

U1 cells or the Patient’s PBMC were stimulated by the indicated concentrations of TLR7/8 agonists, and the supernatants were collected for detection. To measure HIV p24 antigen, the supernatants were diluted into an appropriate concentration and analyzed by HIV-1 ELISA kit (Key-Bio Biotech).

### Western blot

For measuring TLR8, 2 × 10^6^ U1 cells were centrifuged to remove the supernatant, then were incubated with 200 μl RAPI (Beyotime) and 2 μl PMSF (Beyotime) on ice for 30 min. After centrifuging at 16,000 × g 10 min, whole proteins in the supernatant were obtained for western-blot assay.

For measuring NF-kB p65 and phosphor-p65 S536, 5 × 10^6^ U1 cells were stimulated by 1 μM TLR7/8 agonists D018 (7/8), B-130a (8), GS9620 (7), and DMSO (negative control) for 30 min. Nuclear proteins were extracted by NE-PER Nuclear and Cytoplasmic Extraction Reagents (Pierce) and measured the concentration using BCA protein measure kit (Thermo).

Equal amounts of extracts were loaded on the 10% polyacrylamide gel SDS-PAGE. Then proteins were electro-transferred to nitrocellulose membranes 300 mA for 90 min and incubated with blocking buffer (5% nonfat dry milk and 0.1% Tween 20 in TBS) for 1 h at room temperature. NF-kB p65, phosphorylate p65-s529, and PCNA (as the control of nuclear protein) were separately detected by NF-κB p65 Mouse mAb (CST), Phospho-NF-κB p65 Ser536 Rabbit mAb (CST) and PCNA Rabbit mAb (CST) in blocking buffer overnight at 4°C. After washing 5 times in TBST (1 × TBS + 0.1% Tween), the membranes were incubated with anti-mouse IgG (H + L) HRP (Promega) and anti-rabbit IgG (H + L) HRP (Promega) for 1 h at room temperature. After washing 5 times in TBST, membranes were incubated with ECL detection reagents (Bio-red) and then developed in Bio-red imager.

### Cytokine assay

The supernatants from PBMCs culture were collected and stored at-20°C until analysis. For the Multi-cytokines assay, the Legend-plex Human Inflammation Panel 1 kit (Biolegend) was used according to the manual. Thirteen human inflammatory cytokines/chemokines were measured, including IL-1β, IFN-α2, IFN-γ, TNF-α, MCP-1 (CCL2), IL-6, IL-8 (CXCL8), IL-10, IL-12p70, IL-17A, IL-18, IL-23, and IL-33.

For the single-cytokines assay, Human TNF-α, IFN-α, and IFN-γ were separately measured by human TNF-α (1,117,202, DKEWE), IFN-α (1,110,012, DKEWE), IFN-γ (1,110,002, DKEWE) ELISA kit.

### J-Lat cells stimulation

5 × 10^5^ PBMCs from health donors were stimulated by TLR7/8 agonists in 96-well plate for 24 h, then cells were transferred to 96-well round plate and spun down at 2000 rpm for 5 min. The supernatants were collected and cultured with 1 × 10^5^ J-Lat cells in 96-well plate. After 48 h, the cell pellets were centrifuged and washed twice with PBS contained 2% FBS and fixed in 2% formaldehyde for FACS analysis using a BD LSR Fortessa flow cytometer (BD Biosciences). GFP positive cells from the live population, defined by forward FSC/SSC gating, were quantified. 10,000 events per treatment condition were analyzed.

For J-Lat and mDDC co-culture assay, 1 × 10^5^ J-Lat cells were cultured with different amount of mDDC in 96-well plate and then stimulated by TLR7/8 agonists for 48 h. The cell pellets were collected for FACS analysis using a BD LSR Fortessa flow cytometer (BD Biosciences).

### Immune cells staining

To analyze cell surface activation markers, PBMCs were stimulated by 1 μM TLR7/8 agonists or DMSO (as a negative control) for 24 h at 37°C. Then cells were collected and washed in FACS buffer (PBS + 2%FBS). Cells were incubated by fluorescence antibodies at 4°C for 30 min. For T cells, CD3-PE-Cy7 (Biolegend), CD4-PE (BD), CD8-FITC (Biolegend), CD25-BV421 (Biolegend), CD69-BV510 (Biolegend). For NK cells, CD16-PE (Biolegend), CD56-APC (Biolegend), CD69-BV510 (Biolegend), NKG2D-BV421 (Biolegend). For monocytes, CD14-PE-Cy7 (Invitrogen), CD40-APC-Cy7 (Biolegend) HLR-DR-BV510 (Biolegend). For dendritic cells, anti-human lineage cocktail-APC (Biolegend), CD11c-PE-Cy7 (Biolegend), CD80-BV510 (Biolegend), CD86-AF488 (Biolegend).

### Data analysis

Flow cytometry data were analyzed through the FlowJo version10.1. The statistical analysis was performed with Graphpad Prism version 7 or Microsoft Excel, including two-way ANOVA, paired t-test. The correlation analysis was performed with GraphPad Prism version 7.

## Data availability statement

The raw data supporting the conclusions of this article will be made available by the authors, without undue reservation.

## Ethics statement

This study was approved by the Institution Review Board of Tsinghua University. The project number is 20200032. Blood sample from HIV-1-infected patients was collected at Beijing YouAn Hospital of Capital Medical University. The project number is EC-B-031-A01-V9.0. The patients/participants provided their written informed consent to participate in this study.

## Author contributions

XBL and LZ conceived, designed, and supervised the entire study. YL conceived the study and estimated the *in vitro* activity of reversing the HIV-1 latency of the novel TLR7/8 agonists and studied their activation of immune cells. ZW designed and synthesized all the novel TLR7/8 agonists. XYL and YH analyzed the activity and mechanism of reversing the HIV-1 latency of TLR7/8 agonists in U1 and J-LAT cell-lines. JH analyzed the activity of enhancement of immune cells by TLR7/8 agonists. XS, XH, and TZ were responsible for screening and collecting blood samples from HIV-1 infected patients. YL, LZ, and ZW wrote the main manuscript. All authors reviewed and edited the manuscript.

## Funding

The research was supported by grants from the Beijing Municipal Science and Technology Commission (No. D17110700050000) and the National Science and Technology Major Project, Ministry of Science and Technology of China (No. 2018-ZX10302-102).

## Conflict of interest

The authors declare that the research was conducted in the absence of any commercial or financial relationships that could be construed as a potential conflict of interest.

## Publisher’s note

All claims expressed in this article are solely those of the authors and do not necessarily represent those of their affiliated organizations, or those of the publisher, the editors and the reviewers. Any product that may be evaluated in this article, or claim that may be made by its manufacturer, is not guaranteed or endorsed by the publisher.
