# Supplementary material for: Novel TLR7/8 agonists promote activation of HIV-1 latent reservoirs and human T and NK cells
Source: Front Microbiol. 2023 Jan 27;14:1033448. doi: 10.3389/fmicb.2023.1033448 (PMC9911797; doi:10.3389/fmicb.2023.1033448)
Supplement: Supplementary file 1 [file Data_Sheet_1.PDF]

Supplementary

Supplementary Figure 1

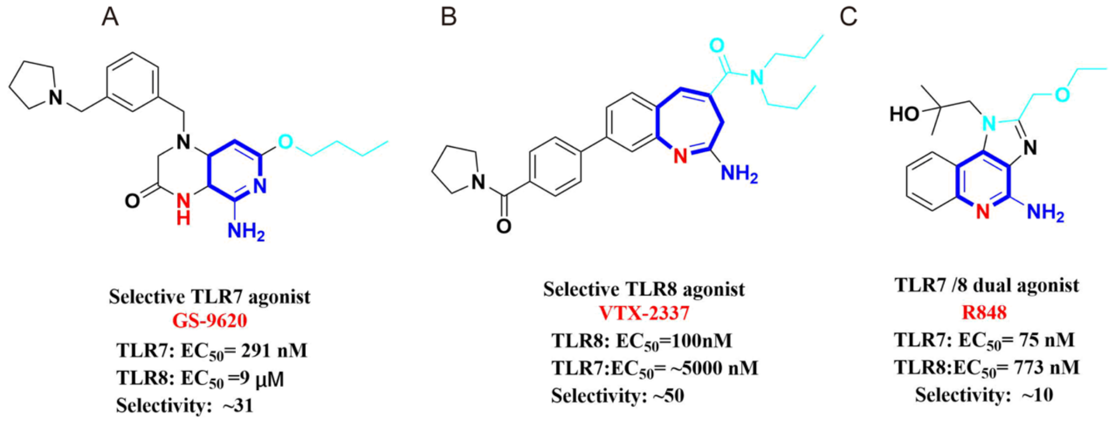

Figure S1. The reported characteristics of commercially available TLR7/8 agonists. The molecular structure, TLR7/8 agonistic activity, and selectivity of commercially available selective or dual TLR7 and TLR8 agonists, including (A)GS9620 (TLR7 agonist), (B)VTX-2337 (TLR8 agonist), and (C) R848 (TLR7/8 dual agonist).

Supplementary Figure 2

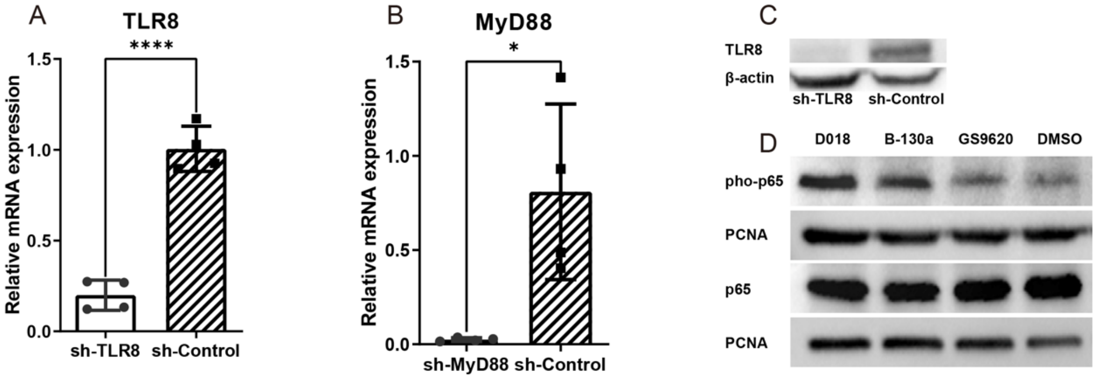

Figure S2. TLR7/8 agonists directly activate latent HIV-1 from the monocytic U1 cell line. (A-B) The relative mRNA expression of (A) TLR8 and (B) MyD88 in TLR8/MyD88 shRNA silenced or control U1 cells. Data are shown as mean ±SD (n=4). (C)TLR8 protein expression in TLR8 shRNA silenced and control U1 cell. (D) The activation of NF-κB in the nucleus of U1 cells stimulated by TLR7/8 agonists was analyzed by western-blot. PCNA is the control for nuclear proteins. Statistical difference between various TLR7/8 agonists was analyzed by two-way ANOVA. ****p<0.0001, ***p<0.001, **p<0.01, *p<0.05, n.s. not significant.

Supplementary Figure 3

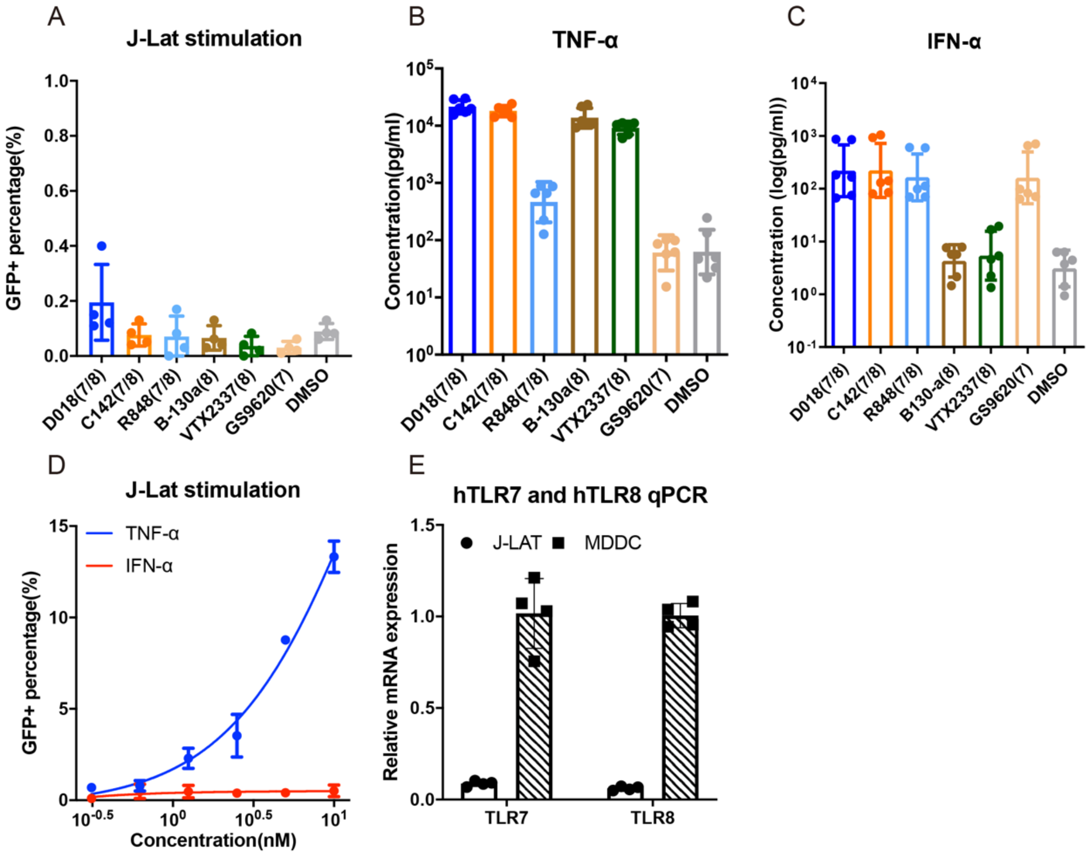

Figure S3. TLR7/8 agonists indirectly activate latent HIV-1 from the J-Lat CD4 T cell line through TNF-α. (A) TLR7/8 agonists activated latent HIV-1 from J-Lat CD4 T cell line directly. Data are presented as mean ±SD (n=4). (B-C) The concentrations of TNF-α (B) and IFN-α (C) in the PBMC supernatants were stimulated by selected TLR7/8 agonists. Data are presented as mean ±SD (n=6). (D) The stimulation of J-LAT by soluble TNF-α and IFN-α. (E) qPCR assays for relative mRNA expression of TLR7 and TLR8 in CD4 T cell-line J-LAT and MDDCs. Statistical difference between various TLR7/8 agonists was analyzed by two-way ANOVA. ****$p<0.0001$, ***$p<0.001$, **$p<0.01$, *$p<0.05$, n.s. not significant.

Supplementary Figure 4

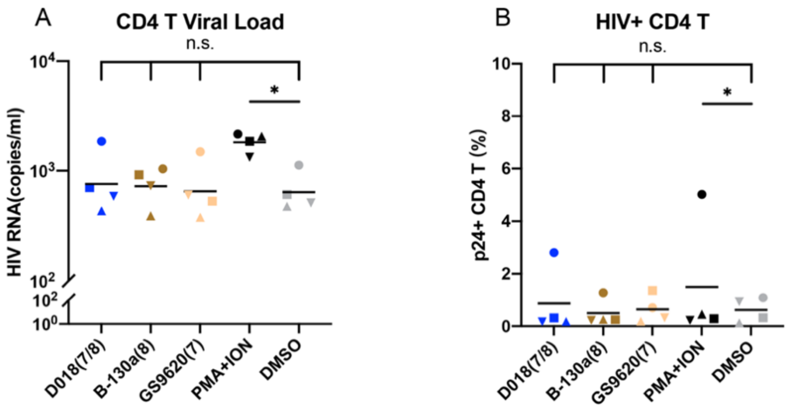

Figure S4. TLR7/8 agonists couldn't activate latent-infected CD4 T cells derived from HIV-1 positive patients. (A-B) The CD4 T cells derived from HIV-1 viremic patients were stimulated by D018 (TLR7/8), B-130a (TLR8), GS9620 (TLR8), PMA plus Ionomycin and DMSO. (A) HIV-1 RNA copies in the supernatant were measured by RT-qPCR and (B) the percentage of p24+ CD4 T cells was analyzed by flow cytometry two days after stimulation(n=4). Statistical difference was analyzed by paired t-test. ****p<0.0001, ***p<0.001, **p<0.01, *p<0.05, n.s. not significant.

Supplementary Figure 5

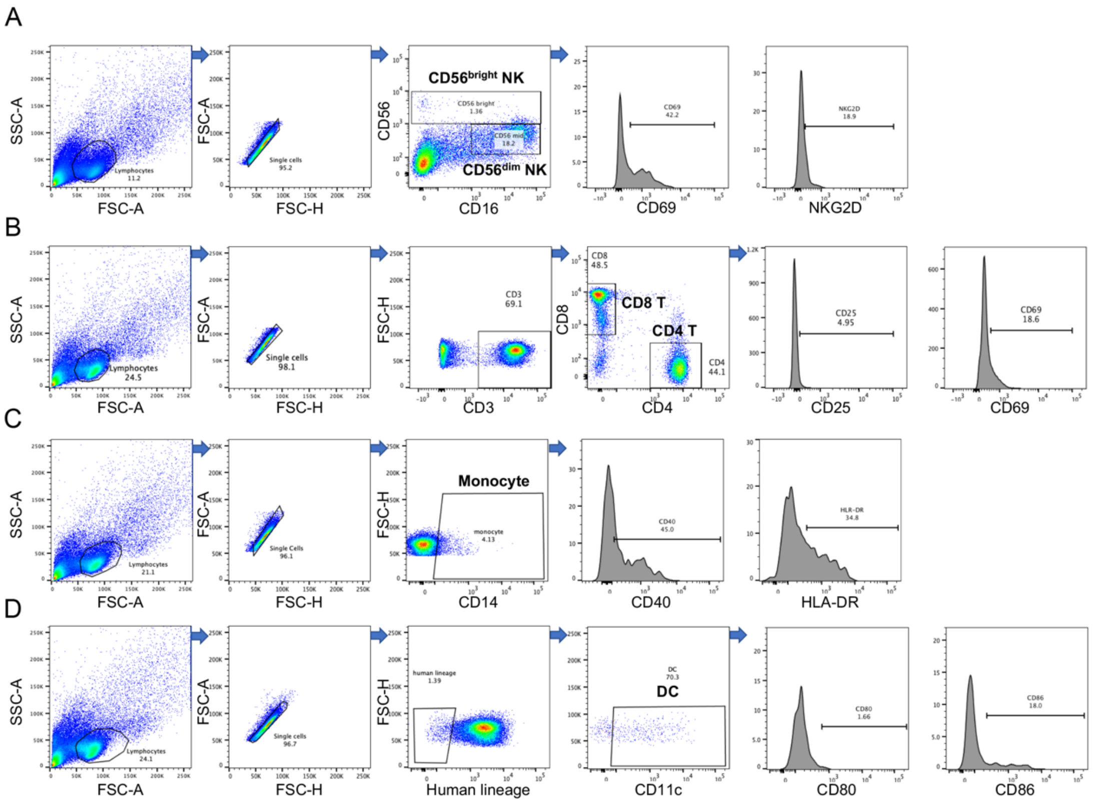

Figure S5. TLR7/8 agonists induce immune cells activation. PBMCs from healthy donors were stimulated by TLR7/8 agonists for 24h and collected to analyze the expression of activation markers of different cell types. (A)The representative diagram of the gating strategy for the expression of CD69 and NKG2D in $CD56^{bright}$ and $CD56^{dim}$ NK cells. (B) The representative diagram of the gating strategy for the expression of CD69 and CD25 in CD4 and CD8 T cells. (C) The representative diagram of the gating strategy for the expression of CD40 and HLA-DR in monocytes. (D) The representative diagram of the gating strategy for the expression of CD80 and CD86 in Dendritic cells.
